# Supplementary material for: A curated dataset and lightweight deep learning framework for tea leaf disease classification
Source: PLoS One. 2026 May 15;21(5):e0349210. doi: 10.1371/journal.pone.0349210 (PMC13178992; doi:10.1371/journal.pone.0349210)
Supplement: Attachment — Submitted filename: Response to Reviewers.pdf [file pone.0349210.s002.pdf]

Reviewer #1: Major Comments

All corrections made in response to comments raised by Reviewer 1 are highlighted in blue in the revised manuscript.

Dataset Size and Generalizability: While the 2000-image dataset is a significant contribution, especially being publicly available and expert-annotated, it is relatively small for training complex deep learning models, particularly when considering the diversity of natural conditions. The underperformance of ResNet50 due to "dataset limitations" hints at this. This raises concerns about the long-term generalizability of the MobileNetV3 model to unseen variations in tea gardens beyond the specific collection sites in Bangladesh.

Response:

We sincerely appreciate the reviewer's insightful observation regarding the dataset size and its impact on model generalizability. To directly address the limitations of the 2,000-image dataset, we fundamentally overhauled our training pipeline. First, we implemented dynamic, on-the-fly data augmentation using the Albumentations framework to artificially expand dataset diversity. This crucial update allowed previously struggling deep networks like ResNet50 to properly converge, improving its accuracy to 95.60%. Second, to ensure robust long-term generalizability beyond the specific collection sites, we replaced the standalone MobileNetV3 with our novel dual-branch Hybrid Feature Fusion architecture, which achieved a peak accuracy of 96.80% without overfitting to regional nuances. Finally, we fully agree with your concern regarding cross-regional generalizability, and we have added a dedicated "Limitations and Future Work" section to the revised manuscript explicitly acknowledging the dataset size constraints and outlining our roadmap for multi-regional and seasonally diverse data collection.

Lack of Detailed Methodology for Model Training: The summaries mention various models and metrics but lack specifics on hyperparameter tuning, training epochs, optimizer choice, learning rate schedules, and cross-validation strategies. For a Q1 journal, these details are crucial for reproducibility and to ensure the reported performance is robust and not a result of specific tuning for MobileNetV3 alone.

Response:

We sincerely appreciate the reviewer for highlighting the need for greater methodological detail to ensure reproducibility and rigorous benchmarking. To directly address this concern, we have comprehensively rewritten the "Training & Validation Setup" section in the revised manuscript to include all precise training parameters and hyperparameters used in our study. Specifically, we detailed our transition to the PyTorch framework, explicitly stating the use of the AdamW optimizer with an initial learning rate of 0.001 for its superior weight decay management. We also specified the standardized use of a batch size of 16, a maximum configuration of 20 epochs, and a strict early stopping protocol with a patience window of 10 epochs based on validation loss monitoring. Furthermore, we clarified our cross-validation and splitting strategy, confirming the use of a stratified 80-10-10 split to perfectly preserve class distributions across all phases. To handle dataset

imbalances, we detailed our integration of dynamically computed class weights and advanced loss functions like Focal Loss and Label Smoothing. Crucially, to ensure our reported performance is robust and not the result of biased tuning for a single model, we explicitly state that this exact standardized hyperparameter configuration was systematically applied across all seven evaluated architectures.

Interpretability (Grad-CAM) Details: While Grad-CAM is mentioned, its application and the insights derived from it are not elaborated. For a Q1 journal, simply stating its use is insufficient; the paper should demonstrate how Grad-CAM contributes to understanding the model's decision-making, identifying critical disease features, or validating expert annotations.

Response:

We sincerely appreciate the reviewer for their critical observation regarding the application of Grad-CAM and the lack of derived insights. The reviewer is entirely correct that simply mentioning the tool without providing detailed visualizations and agronomic validation is insufficient for a rigorous analysis. We must transparently clarify that Grad-CAM was prematurely mentioned in our initial draft; the interpretability pipeline has not yet been fully implemented or trained in the current scope of this study. To correct this error and maintain the paper's integrity, we have removed the mention of Grad-CAM from the Abstract. Furthermore, following the reviewer's excellent point about the critical importance of understanding the model's decision-making process, we have explicitly incorporated the application of explainable AI techniques, such as Grad-CAM, into the "Limitations and Future Work" section of the revised manuscript. We plan to dedicate a comprehensive future study specifically to the visual interpretation and expert validation of our proposed hybrid architecture's feature extraction capabilities.

Real-time Application Claims: The paper frequently mentions "real-time" and "resource-constrained" applications. While MobileNetV3 is designed for this, the summaries do not provide any empirical evidence (e.g., inference time on target hardware, model size comparison) to support these claims beyond its architectural design.

Response:

We sincerely appreciate the reviewer for their critical observation regarding the empirical validation of our real-time application claims. The reviewer is absolutely correct that claiming true real-time operational status requires concrete empirical hardware testing, such as inference time on target edge devices, which was lacking in our initial manuscript. To address this, we have carefully revised our phrasing throughout the new manuscript to clarify that our current study provides a theoretical and architectural foundation that is highly optimized for such environments, rather than a physically deployed system. We now frame the proposed Hybrid Feature Fusion model which leverages the inherently lightweight MobileNetV3 and EfficientNetV2-Small backbones as an "ideal candidate" with strong "potential" for real-time IoT integration. Furthermore, we have explicitly designated physical hardware deployment and empirical benchmarking as the next critical phase of our research. We have added an "IoT Integration" point in the newly created "Limitations and Future Work" section, stating our objective to deploy and evaluate the model on IoT-enabled mobile and edge devices for real-time field monitoring. We believe our current findings offer a highly suitable and accurate framework that will serve as an excellent baseline for these future practical implementations.

Minor Comments

Clarity on "Custom CNN": The summaries mention a "custom CNN" alongside pre-trained models. Its architecture, performance, and comparison against the pre-trained models are not detailed, making it difficult to assess its contribution or baseline performance.

Response:

The reviewer is absolutely correct that without a detailed architectural breakdown and a transparent performance baseline, the contribution of the custom model could not be properly assessed. To fully address this, we have introduced a dedicated "Custom CNN Model Architecture and Implementation" section in the revised manuscript, which explicitly details the network's six sequential convolutional blocks, channel expansions, activation functions, pooling layers, and custom classification head. Furthermore, we expanded the "Models Evaluation Results" section to include a comprehensive analysis of the Custom CNN's training dynamics, where it achieved a peak validation accuracy of 89.60% and a validation Area Under the Curve (AUC) of 0.9766. Finally, to ensure clear baseline comparisons against the pre-trained networks, the Custom CNN has been fully integrated into our comparative benchmarking tables alongside all evaluated architectures, demonstrating its role as a solid foundational baseline for our study.

Specifics of Preprocessing: While CLAHE, brightness adjustment, and unsharp masking are listed, the specific parameters or rationale for choosing these particular techniques are not provided.

Response:

We sincerely appreciate the reviewer for pointing out the need for greater detail regarding our preprocessing methodology. The reviewer is absolutely correct that listing techniques without their specific parameters and rationale makes it difficult to fully understand their impact. To address this, we have expanded the 'Image Pre-Processing and Data Augmentation' section in the revised manuscript to explicitly detail both the rationale and the exact parameters used in our pipeline.We clarify that our preprocessing focuses on architectural compatibility and gradient stability through standardized resizing to 224 X 224 pixels and ImageNet-based normalization (mean: [0.485, 0.456, 0.406], std: [0.229, 0.224, 0.225]) . Regarding the pixel-level enhancements, we utilize stochastic brightness and contrast adjustments (applied with a 20% probability) to specifically mitigate the effects of variable outdoor illumination and shadows encountered during field collection . Furthermore, we detail the parameters of our spatial transformations, such as random rotations of up to 15 degrees (70% probability) and horizontal flips (50% probability), which were chosen to ensure the networks learn to recognize pathological symptoms regardless of leaf orientation or camera angle. These comprehensive details and their underlying rationales have been seamlessly integrated into the 'Image Pre-Processing and Data Augmentation' section of the revised manuscript.

Dataset Class Distribution: The summaries do not mention the class distribution (Blight, Red Rust, Helopeltis, Healthy). An imbalanced dataset could lead to inflated accuracy metrics, and specific strategies to handle imbalance (if any) should be discussed.

Response:

We sincerely appreciate the reviewer for raising this critical point regarding class distribution and the potential for inflated accuracy metrics due to dataset imbalance. In the revised manuscript, we have explicitly clarified that the 2,000-image dataset is evenly distributed across the four target classes (Blight, Red Rust, Helopeltis, and Healthy) to naturally establish a balanced baseline. Furthermore, to rigorously address any residual micro-imbalances and guarantee that our reported accuracy and AUC metrics are not inflated, we have comprehensively detailed our mitigation strategies in the updated text. Specifically, we implemented a stratified 80-10-10 dataset split to perfectly preserve the exact class distribution across the training, validation, and testing phases. Crucially, we also upgraded our training pipeline to dynamically compute balanced class weights during runtime. As explicitly stated in the revised text, these weights are integrated directly into the Cross-Entropy loss function, functioning alongside advanced mechanisms like Focal Loss and Label Smoothing to robustly penalize majority class bias.

Environmental Conditions: The dataset was collected "under natural conditions." More details on the variability of these conditions (lighting, background clutter, leaf orientation, disease severity stages) would strengthen the methodology.

Response:

We sincerely appreciate the reviewer for their valuable feedback regarding the environmental conditions of our dataset. The reviewer makes an excellent point that simply stating the data was collected under "natural conditions" does not provide enough methodological transparency regarding the actual challenges the models must overcome. To directly address this, we have significantly expanded the "Data Collection" section in the revised manuscript to explicitly detail the environmental variability captured within the dataset. We now clearly state that the images encompass a wide range of lighting conditions (including direct sunlight, overcast skies, and canopy shading) and feature realistic background clutter such as overlapping foliage and branches. Furthermore, we explicitly mention that the leaves were photographed from multiple orientations and capture the targeted diseases across various stages of severity, from early microscopic spots to advanced structural deformation. By outlining these specific complexities, we aim to better highlight the robustness of our proposed Hybrid Feature Fusion model in isolating genuine pathological symptoms from noisy, real-world agricultural backgrounds. These updates have been fully integrated into the "Data Collection" section of the revised manuscript.

Weaknesses and Limitations

Dataset Size for Generalization: As noted in major comments, 2000 images, while valuable, might be insufficient to capture the full spectrum of disease variations and environmental conditions, potentially limiting the model's generalizability.

Response:

To address this highly valid concern directly in the revised manuscript, we have implemented a three-tiered response. First, we explicitly acknowledged this dataset scale constraint in our expanded "Limitations and Future Work" section. Second, we updated the "Data Collection" section to clarify our dataset curation philosophy: rather than scraping massive amounts of noisy, imbalanced data, we prioritized class equilibrium , high-variance natural field conditions, and strict multi-stage expert diagnostic fidelity over sheer volume. Third, and most importantly, to definitively prove that our model's generalizability is not artificially restricted by this dataset size, we added a new "Cross-Dataset Generalization" subsection to the Results. In this section, we evaluated our trained model on an entirely unseen, external public benchmark (teaLeafBD).

Limited Methodological Detail: Insufficient detail on training parameters, hyperparameter tuning, and specific preprocessing parameters hinders reproducibility.

Response:

To directly address this and ensure complete transparency, we have significantly expanded our methodological descriptions in the revised manuscript. First, in the updated 'Image Pre-Processing and Data Augmentation' section, we now explicitly define the standardized parameters for our data pipeline, including resizing to 224 X 224 pixels, ImageNet-based normalization, and the exact execution probabilities for our dynamic Albumentations suite—such as random rotations (70%), horizontal flips (50%), and brightness/contrast adjustments (20%). Second, we have introduced a dedicated 'Hyperparameter Configuration and Training Protocol' subsection. This new section explicitly details our entire experimental environment and training pipeline, specifying the use of the AdamW optimizer for enhanced weight decay management, an initial learning rate of 0.001, a batch size of 16, and our precise early-stopping criteria with a patience of 10 epochs based on validation loss monitoring . We believe these comprehensive details provide a robust framework for the reproducibility of our findings across the eight evaluated architectures.

Lack of Empirical Evidence for Real-time Claims: The paper claims real-time applicability but lacks concrete benchmarks (e.g., inference speed, memory footprint) on actual edge devices.

Response:

Because this initial study focuses primarily on the curation of the balanced dataset and the theoretical development of the dual-branch Hybrid Feature Fusion architecture, comprehensive hardware-specific edge benchmarking falls outside our current scope. To directly address the

reviewer's highly valid concern and ensure we do not overstate our empirical findings, we have tempered our claims throughout the revised manuscript. Furthermore, we have explicitly moved the physical deployment and benchmarking of the model into the newly expanded "Limitations and Future Work" section. The revised text now formally outlines "IoT Integration" as a primary future objective, specifically noting our plans to deploy and rigorously test the model on IoT-enabled mobile and edge devices for real-time field monitoring.

Under-explanation of ResNet50's Underperformance: While attributed to "dataset limitations," a deeper analysis of why a more complex model struggled would be beneficial. Was it overfitting, or simply too large for the feature complexity present in the dataset?

Response:

To directly address this, we completely overhauled our methodology for the revised manuscript. As detailed in the newly added "ResNet50 Model Architecture and Implementation" section, we discarded the default fully connected layer and engineered a custom Multi-Layer Perceptron (MLP) head. By strategically integrating 1D batch normalization to stabilize deep feature transitions and applying aggressive dropout rates (40% and 20%) to actively mitigate overfitting, we successfully unlocked the architecture's true potential. We are pleased to report that under this new, rigorously controlled hyperparameter protocol, ResNet50 no longer underperforms; it achieved an outstanding peak validation accuracy of 95.60% and an AUC of 0.9975.

Absence of Ablation Studies: No mention of ablation studies to justify the choice of specific preprocessing steps or data augmentation techniques.

Response:

To directly address this and mathematically prove the value of our pipeline, we have added a new 'Ablation Study on Preprocessing and Augmentation' subsection to the Discussion in the revised manuscript . We conducted an empirical analysis using our proposed Hybrid Feature Fusion architecture, evaluating its performance under two distinct configurations: an unoptimized baseline and our fully configured pipeline. The revised text explicitly details how the baseline configuration, despite reaching high raw accuracy, struggled with extreme learning instability and performance volatility, characterized by a validation loss spike of 4.8279. In contrast, our fully configured pipeline—incorporating the complete Albumentations suite (rotations, horizontal flips, and brightness adjustments)—delivered superior convergence stability and a peak macro AUC of 0.9980 . This empirical comparison explicitly justifies our methodological choices, proving that the integration of dynamic spatial and pixel-level augmentations is strictly necessary to maximize the feature extraction stability of the dual-branch network.

Specific and Actionable Recommendations for Improvement

Expand Dataset Analysis: Provide a detailed breakdown of the dataset, including class distribution, examples of images for each class, and a discussion on the diversity of conditions (lighting, background, disease severity).

Response:

To directly address this, we have significantly expanded both the "Data Collection" and "Image Pre-Processing and Data Augmentation" sections in the revised manuscript. First, we have added detailed summary tables that explicitly outline the dataset's perfectly balanced class distribution and its strict stratified 80-10-10 split. Second, we incorporated a comprehensive new visual figure that provides sample images while simultaneously demonstrating the stage-by-stage progression of our data pipeline from raw capture through augmentation. Finally, we substantially expanded the textual discussion surrounding dataset diversity; the revised text now explicitly details our targeted capture of high-variance natural conditions, specifically noting the inclusion of dynamic lighting (midday sun, overcast, canopy shading), complex background clutter, and various stages of disease severity ranging from early-stage minute necrotic spots to advanced structural degradation.

Detail Training Methodology: Include a dedicated section or subsection detailing the training setup for all models: optimizer, learning rate schedule, batch size, number of epochs, data split strategy (e.g., k-fold cross-validation), and hyperparameter tuning approach.

Response:

To directly address this, we have comprehensively reorganized and expanded the relevant sections in the revised manuscript. First, under the "Training & Validation Setup" section, we have clearly outlined our data split strategy, including an explicit table demonstrating the stratified 80-10-10 split utilized to guarantee class equilibrium. Furthermore, we have introduced a dedicated "Hyperparameter Configuration and Training Protocol" subsection. This new section serves as a centralized breakdown of our exact training setup. It explicitly details the use of the AdamW optimizer, a 0.001 initial learning rate, a fixed batch size of 16, and our precise early-stopping criteria capped at 20 epochs. Additionally, we have detailed the empirical grid-search approach utilized during the baseline phase to tune these specific hyperparameters.

Quantify Real-time Performance: Conduct experiments to measure inference time and model size on representative edge computing hardware (e.g., Raspberry Pi, NVIDIA Jetson) to empirically support claims of real-time and resource-constrained applicability.

Response:

Because this initial study was designed strictly to curate the balanced tea leaf dataset and develop the theoretical foundation of the dual-branch Hybrid Feature Fusion architecture, comprehensive

hardware-specific edge benchmarking falls outside the boundaries of our current experimental setup.

Elaborate on Grad-CAM Insights: Present and discuss specific Grad-CAM visualizations. Show how they highlight relevant disease features and confirm the model's focus on diagnostically important regions or reveal any spurious correlations.

Response:

Because this foundational study was primarily focused on curating the perfectly balanced dataset and establishing the quantitative accuracy benchmarks for the new dual-branch Hybrid Feature Fusion architecture, comprehensive XAI visualization was not included in the initial experimental pipeline. To address the reviewer's highly valid point and ensure our manuscript's claims accurately reflect the data presented, we have removed mentions of completed Grad-CAM analysis from the revised text. Furthermore, we have explicitly added "Model Interpretability" into our newly expanded "Limitations and Future Work" section.

Analyze ResNet50's Performance: Provide a more in-depth analysis of why ResNet50 underperformed. This could involve analyzing its training curves (loss, accuracy), examining Grad-CAM outputs for ResNet50, or discussing the potential for overfitting given the dataset size.

Response:

To directly address this, we completely overhauled our methodology for the revised manuscript. As detailed in the newly added "ResNet50 Model Architecture and Implementation" section, we discarded the default fully connected layer and engineered a custom Multi-Layer Perceptron (MLP) head. By strategically integrating 1D batch normalization to stabilize deep feature transitions and applying aggressive dropout rates (40% and 20%) to actively mitigate overfitting, we successfully unlocked the architecture's true potential. Under this new, rigorously controlled hyperparameter protocol, ResNet50 achieved an outstanding peak validation accuracy of 95.60% and an AUC of 0.9975.

Consider Ablation Studies: If feasible, perform ablation studies on preprocessing techniques or data augmentation strategies to demonstrate their individual contributions to the model's performance.

Response:

To address this, we have incorporated a dedicated Ablation Study section in the revised manuscript to quantitatively demonstrate the necessity of each component.

Discuss Limitations of Dataset Size: Explicitly acknowledge the limitations of the current dataset size regarding generalizability and discuss strategies for future expansion.

Response:

In the revised manuscript, we have explicitly acknowledged that a dataset of 2,000 images, while expertly annotated and strictly balanced, represents a moderate sample size that may not fully encompass the vast environmental variability found in global tea plantations. To address concerns regarding generalizability, we have introduced a new "Cross-Dataset Generalization" subsection, where we validated our Hybrid Feature Fusion architecture against the independent teaLeafBD benchmark . This evaluation demonstrates that our dual-branch approach successfully learns robust pathological features that remain effective across different camera sensors and background complexities.

Refine "Custom CNN" Description: Provide a clear architectural description and performance metrics for the "custom CNN" to establish a baseline.

Response:

In the revised manuscript, we have included a comprehensive architectural breakdown of the Custom CNN to serve as a transparent performance baseline. As detailed in the "Custom CNN Model Architecture and Implementation" section.

Reviewer #2:

All corrections made in response to comments raised by Reviewer 2 are highlighted in yellow in the revised manuscript.

- The preprocessing was heavy; it must be justified.

Response:

We sincerely appreciate the reviewer for highlighting this important point. The reviewer is absolutely correct that a preprocessing pipeline must have clear scientific justification to avoid introducing artificial artifacts. In our initial draft, we failed to properly explain the necessity of these steps. To directly address this, we have comprehensively expanded the 'Image Pre-Processing and Data Augmentation' section in the revised manuscript to justify our methodology. Because the dataset was collected under highly variable natural field conditions—including harsh midday sunlight, deep canopy shadows, and complex background clutter—the raw images suffered from severe illumination and scale inconsistencies . We explicitly clarify in the revised text that standardized ImageNet-based normalization and resizing were strictly required to ensure stable gradient convergence and architectural compatibility across the seven evaluated models . Furthermore, we justified the use of a dynamic augmentation pipeline (via Albumentations), specifically including stochastic brightness and contrast adjustments . These steps were mandatory to mitigate the effects of variable outdoor lighting and ensure that the dual-branch network could successfully isolate fine-grained symptomatic boundaries from noisy, real-world agricultural backgrounds . We believe this level of preprocessing provides a robust and balanced foundation for accurate feature extraction.

- I don’t know whether the data augmentation has been applied only to the training data or to the entire dataset. In fact, only training data can be augmented.

Response:

The reviewer is absolutely correct that data augmentation must strictly be confined to the training dataset to prevent data leakage and invalid evaluation metrics. We understand how the phrasing in our initial manuscript could have caused ambiguity regarding when and where the augmentations were applied. To completely eliminate any confusion, we have explicitly clarified this process in the revised manuscript. We now detail that after the initial 80-10-10 dataset split, we implemented a dynamic, on-the-fly data augmentation strategy utilizing the Albumentations library exclusively during the training phase. We have added explicit statements confirming that while the training set is subjected to stochastic spatial and pixel-level transformations, the validation and testing subsets remain strictly unaugmented. This ensures a completely fair and unbiased evaluation of the models.

- The presentation of the pretrained algorithms used can be improved by highlighting only their deployment for the target task.

Response:

The reviewer is entirely correct that providing generic, textbook descriptions of established architectures distracts from the actual methodological contributions of the study. In our initial manuscript, the model descriptions were overly broad and lacked specific details regarding their adaptation to our dataset. To directly address this, we have completely overhauled the "Compared Models" section in the revised manuscript. We removed the generic background filler and focused exclusively on how these pre-trained backbones were structurally modified and deployed for our specific target task. By directly tracking our implementation code , we explicitly detail in the revised text how the native classification heads of each pre-trained model including ResNet50, DenseNet121, MobileNetV3, EfficientNetV2-B3, and ViT-B16 were entirely discarded. We meticulously describe the custom-engineered multi-layer perceptron (MLP) heads we integrated in their place to map the extracted features directly to our four-target tea leaf disease classes. For example, the revised text now outlines the specific linear projections, activation functions (such as Hardswish for MobileNetV3 and GELU for the Vision Transformer), 1D batch normalization steps, and targeted dropout rates utilized to adapt these generalized feature extractors to our specific dataset.

- The structure of the standard CNN must be described in detail.

Response:

The reviewer is absolutely correct that the generic description and mathematical formulas provided in our initial manuscript were insufficient for proper architectural comparison or reproducibility. To completely resolve this, we have replaced the generic definitions with a dedicated "Custom CNN Model Architecture and Implementation" section in the revised manuscript. In this new section, we explicitly detail the exact layer-by-layer construction of our custom-built network directly from our codebase. We describe the feature extraction backbone, which consists of six sequential convolutional blocks, specifying the use of 3x3 kernels, zero-padding of 1, and the precise channel expansions from an initial 32 filters to 64 filters across the subsequent five layers. Furthermore, we outline the integration of in-place ReLU activations, 2x2 max pooling operations, Kaiming normal weight initialization, and the exact configuration of the adaptive average pooling and 64-unit fully connected classification head.

- ResNet50 achieves only 53% accuracy, which is unusually low. This result must be well justified. This poor performance can result from an unsuitable finetuning. Also, a larger ResNet can be experimented with.

Response:

The reviewer's intuition was absolutely correct; the poor performance of 53% was indeed the result of an unsuitable fine-tuning strategy and an unoptimized classification head, rather than an inherent limitation of the architecture itself. To directly address this, we completely overhauled our transfer learning pipeline for ResNet50. As detailed in the revised manuscript, we discarded the default fully connected layers and engineered a custom Multi-Layer Perceptron (MLP) head. This new classification mechanism incorporates 1024- and 512-dimensional projections, in-place ReLU activations, 1D batch normalization to stabilize deep feature transitions, and strategic dropout rates of 40% and 20% to mitigate overfitting. With this optimized fine-tuning approach and the integration of the AdamW optimizer, ResNet50's performance improved dramatically, achieving an impressive peak validation accuracy of 95.60%. Because the network now successfully captures the complex spatial hierarchies of the tea leaf diseases and generalizes exceptionally well.

- The dataset annotations must be well explained, as any misannotation will introduce bias.

Response:

The reviewer is correct that a vague explanation of the labeling process leaves the dataset vulnerable to questions of reliability and subjective misclassification. To directly address this, we have significantly expanded the "Data Collection" section in the revised manuscript to explicitly detail our rigorous annotation protocol. We now clarify that the images were independently inspected by multiple agricultural domain experts specializing in plant pathology, utilizing strictly defined phenotypic disease markers for Blight, Red Rust, and Helopeltis. Furthermore, we explicitly outline our consensus mechanism, detailing that any diagnostic disagreements were resolved by a third senior agriculturalist to eliminate subjective bias. By providing these specific methodological safeguards, we ensure the high fidelity of the ground truth labels before they enter the preprocessing and training pipelines.

- At least for the best two algorithms, show the confusion matrix and the classification report.

Response:

The reviewer is absolutely correct that providing the confusion matrices and full classification reports is essential for a granular understanding of where the models excel and where they struggle with specific class misclassifications. To keep the main manuscript concise while fully addressing this requirement, we have generated these comprehensive metrics for our architectures. The high-resolution confusion matrices, alongside the complete classification reports detailing the precision,

recall, and F1-scores for each individual disease category, have been compiled and are now fully accessible in the Supporting Information document accompanying the revised manuscript.

- A transformer-based model, such as ViT, can be considered.

Response:

We sincerely appreciate the reviewer for their excellent suggestion to consider a transformer-based model. The reviewer is absolutely correct that exploring non-convolutional paradigms like Vision Transformers provides a much more comprehensive and rigorous architectural evaluation. In our initial manuscript, our benchmarking was strictly limited to CNN-based models. To directly address your recommendation, we have fully integrated the Vision Transformer (ViT-B16) into our revised study.

- In the abstract authors mentioned the following:" Furthermore, Gradient-Weighted Class Activation Mapping (Grad-CAM) was utilized to visualize and interpret the features influencing predictions" . This part was not included in the paper, so it is better to remove it from the abstract. Alternatively, it could be a good addition to include Grad-CAM visualization.

Response:

The reviewer is correct that our initial abstract mistakenly claimed the inclusion of Grad-CAM visualizations, which were not actually part of the experimental scope or results of the study. To rectify this and ensure complete transparency, we followed your first recommendation and completely removed the mention of Grad-CAM from the Abstract in the revised manuscript. However, we completely agree with your second point that visualizing network attention is a highly valuable addition for this domain. Therefore, instead of abandoning the concept, we have explicitly designated the integration of Explainable AI (XAI) techniques specifically Grad-CAM as a primary research objective in our newly expanded "Limitations and Future Work" section. We note that implementing this in the future will be critical to visually validating disease features and building agronomic trust.

Reviewer #3:

All corrections made in response to comments raised by Reviewer 3 are highlighted in green in the revised manuscript.

1. Limited Methodological Novelty: The theoretical novelty of the work appears limited, as MobileNetV3-based architectures for similar plant disease classification tasks have been extensively explored in recent literature. For instance, the authors should review and differentiate their work from existing studies such as
ohttps://doi.org/10.3389/fpls.2024.1459292
ohttp://doi.org/10.14569/ijacsa.2024.0150560
ohttps://doi.org/10.1371/journal.pone.0323322
ohttps://doi.org/10.1186/s13007-025-01452-y
ohttps://doi.org/10.11975/j.issn.1002-6819.202308149

Response:

We sincerely appreciate the reviewer for directing our attention to these recent studies and for their critical observation regarding the limited methodological novelty of standalone MobileNetV3 applications. The reviewer is absolutely correct that MobileNetV3 architectures have been extensively explored in recent literature, making a basic implementation theoretically insufficient. This precise limitation is exactly what drove the major architectural overhaul in our revised manuscript. As detailed in the updated text, we no longer propose a standalone MobileNetV3 model. Instead, we have developed a novel Hybrid Feature Fusion Architecture. To properly contextualize our advancement, we have incorporated all five of the reviewer's suggested references into the Introduction section. We explicitly differentiate our work by highlighting that while the recent studies primarily optimize single-branch MobileNet architectures via attention mechanisms or frequency-domain fusions (such as 2D DWT), our methodology utilizes a dual-spatial-branch approach. By running EfficientNetV2-Small and MobileNetV3-Small in parallel, our hybrid model uniquely overcomes single-branch limitations by simultaneously capturing both fine-grained local textures and global structural degradation.

2. To demonstrate the robustness and generalizability of the proposed model, the study must be evaluated on other established benchmarks (e.g., TDPD, Tea_Leaf_Disease, and teaLeafBD). A comparative analysis between the performance on these datasets and your newly curated dataset is essential.

Response:

We sincerely appreciate the reviewer for their critical suggestion to evaluate our model on external benchmarks. The reviewer is absolutely correct that demonstrating high accuracy on a single, newly curated dataset is insufficient to prove true architectural robustness, as the model could simply be overfitting to specific dataset artifacts. To directly address this limitation and prove

generalizability, we have expanded our experimental scope and added a new "Cross-Dataset Generalization" subsection within the Discussion section of the revised manuscript. We evaluated our proposed Hybrid Feature Fusion model on the established teaLeafBD benchmark dataset, which introduces entirely different environmental conditions, lighting, and background clutter. Utilizing the exact same training protocols, our model demonstrated exceptional adaptability, achieving highly competitive accuracy and AUC on the external benchmark. This comparative analysis explicitly confirms that our dual-branch architecture learns genuine, generalizable pathological features rather than dataset-specific noise. We thank the reviewer for this recommendation, as it has significantly strengthened the empirical validation of our proposed framework.

3. Provide a comprehensive comparison between the newly curated dataset and existing state-of-the-art (SOTA) tea leaf datasets. This analysis should explicitly outline the strengths, weaknesses, and specific gaps addressed by your data.

   ## Response:

   We sincerely appreciate the reviewer for their excellent suggestion to provide a comparative analysis of our dataset against existing state-of-the-art collections. The reviewer is correct that outlining the specific strengths, weaknesses, and addressed gaps is essential for contextualizing the scientific contribution of our newly curated data. To directly address this, we have expanded the "Data Collection" section in the revised manuscript to include a comprehensive comparison against established benchmarks like teaLeafBD and TDPD. We explicitly outline that while our dataset is moderately sized and limited to four classes (which we acknowledge as a weakness compared to massive, multi-class datasets), its primary strengths lie in its class equilibrium, multi-expert diagnostic fidelity, and high-variance natural environmental conditions. We highlight that by prioritizing these qualitative metrics over sheer volume, our dataset specifically addresses the existing gap of high-fidelity, perfectly balanced benchmarks needed to train field-ready models without the bias introduced by synthetic oversampling. This critical analysis has been fully integrated into the revised text to clearly demonstrate the dataset's unique value to the research community.

4. Crucial details regarding the dataset capturing process are currently missing. Please specify the hardware used (e.g., camera specifications, focal length, lighting conditions) and the environmental settings during image acquisition.

   ## Response:

   The reviewer is correct that documenting the specific hardware and environmental parameters is essential for evaluating the dataset's quality and reproducibility. To directly address this, we have incorporated the requested specifics into the "Data Collection" section of the revised manuscript. We now explicitly state that the dataset was acquired using an iPhone 12 Pro Max equipped with a 12 MP triple-camera system, capturing high-resolution images ranging from 1080x1080 to 3024x3024 pixels to ensure the retention of fine-grained pathological textures.

5. The manuscript lacks visual representation of the data. Please include:
    - Sample images showing raw, pre-processed, and augmented image samples from dataset.
    - A detailed summary table describing the dataset (e.g., class distribution, resolution, and total samples per category).

    Response:

    The reviewer is correct that explicitly illustrating the dataset composition and the transformations it undergoes is essential for methodological clarity. To directly address this, we have incorporated a new, comprehensive visual figure (Figure 3) into the revised manuscript that clearly demonstrates the stage-by-stage progression of our image pipeline, showcasing raw field captures, their standardized states post-resizing (224×224 pixels), and their final augmented states. Furthermore, we have added detailed summary tables that explicitly outline the dataset's attributes. Table 1 provides a breakdown of the balanced class distribution across the four categories (Blight, Red Rust, Helopeltis, and Healthy), while Table 2 details the strict stratified 80-10-10 split utilized for the training, validation, and testing phases. These additions, combined with the technical rationale for our dynamic augmentation choices, significantly enhance the transparency and reproducibility of our study.

6. It is noted that different models were trained using a varying number of epochs. This leads to an unfair comparison and undermines the validity of the results. To ensure a rigorous benchmarking process, the authors must use a consistent set of hyperparameters (epochs, learning rate, optimizer, etc.) across all models.

    Response:

    To directly address this and ensure complete methodological rigor, we have added a dedicated "Hyperparameter Configuration and Training Protocol" subsection to the revised manuscript. We explicitly clarify that all models were subjected to a strictly standardized training pipeline: a maximum limit of 20 epochs, a uniform batch size of 16, and the AdamW optimizer with a fixed initial learning rate of 0.001. We further explain that any variation in the actual number of epochs trained by each model was strictly the result of a universally applied Early Stopping callback (configured with a patience of 10 epochs) designed to halt training upon convergence and prevent overfitting. By ensuring that the foundational training constraints and optimization mechanics were identical across all architectures, we guarantee a fair and mathematically rigorous comparative analysis.

7. There is insufficient information regarding the experimental environment. Please provide details on the hardware infrastructure (CPU/GPU), total training time, and the specific strategy used for hyperparameter tuning.

Response:

To directly address this, we have expanded the "Hyperparameter Configuration and Training Protocol" section in the revised manuscript to include these precise details. We have explicitly stated the exact hardware infrastructure utilized for the experiments (an NVIDIA RTX 3060 6GB GPU and an AMD Ryzen 7 5700X CPU with 16GB RAM configured with CUDA acceleration), alongside the total convergence time of approximately one hour for the proposed Hybrid model. Furthermore, we detailed our empirical grid-search tuning strategy, clarifying how we evaluated various learning rates and batch sizes to definitively select our optimal configuration (AdamW, LR 0.001, Batch 16) based on validation loss minimization and AUC maximization.

8. The parameters used for data augmentation (e.g., rotation ranges, brightness scales, etc.) must be clearly specified. Furthermore, it is highly recommended that the authors upload the augmented dataset as part of their contribution to enhance transparency and reproducibility.

Response:

To directly address this, we have expanded the "Image Pre-Processing and Data Augmentation" section in the revised manuscript to clearly specify the exact transformations applied via the Albumentations library. The text now explicitly details that our pipeline utilizes random rotations of up to 15 degrees with a 70% execution probability, horizontal flips with a 50% probability, and random brightness and contrast adjustments with a 20% probability. Regarding the recommendation to upload the augmented dataset, we wish to clarify that our methodology employs dynamic, on-the-fly augmentation during the training loop rather than generating a static augmented dataset on disk. To ensure complete transparency and absolute reproducibility, we have made our , raw 2000-image dataset publicly available, and we have uploaded our exact PyTorch data-loading scripts (which contain the deterministic augmentation pipelines) to our public GitHub repository. This ensures any researcher can perfectly replicate our exact training conditions without the massive storage bloat of static augmented files.

9. Given that the study introduces a 'newly curated dataset, the lack of a standardized annotation protocol raises concerns regarding the validity of the ground-truth labels.

Response:

We sincerely appreciate the reviewer for raising this critical point regarding the validity of our ground-truth labels. The reviewer is correct that introducing a newly curated dataset without a transparent, standardized annotation protocol leaves the entire study vulnerable to questions of subjective bias and misclassification. To directly address this and ensure complete methodological rigor, we have significantly expanded the "Data Collection" section in our revised manuscript to detail our strict, multi-stage annotation protocol. We now explicitly outline that all 2,000 images were independently inspected by agricultural domain experts specializing in plant pathology.
