# Supplementary material for: A curated dataset and lightweight deep learning framework for tea leaf disease classification
Source: PLoS One. 2026 May 15;21(5):e0349210. doi: 10.1371/journal.pone.0349210 (PMC13178992; doi:10.1371/journal.pone.0349210)
Supplement: Attachment — Submitted filename: Response_to_Reviewers_auresp_2.pdf [file pone.0349210.s003.pdf]

Reviewer #1

All corrections made in response to comments raised by Reviewer 1 are highlighted in blue in the revised manuscript.

1. Originality and Novelty Contribution: The paper presents a Hybrid Feature Fusion architecture that utilizes EfficientNetV2-S and MobileNetV3-S in parallel. This dual-branch approach is designed to capture both fine-grained local textures (via EfficientNet) and global structural context (via MobileNet), addressing a key limitation in single-branch networks. Novelty: While the individual models are established, their specific hybrid integration for tea leaf disease is novel. The creation of a curated, expertly annotated dataset of 2,000 images from real-world field conditions in Bangladesh adds significant value to the niche field of tea pathology.

   Response:

   We sincerely appreciate the reviewer for their encouraging comments and for recognizing the originality and novelty of our research.

2. Relevance and Significance Topic Relevance: The work is highly relevant to current trends in precision agriculture and the deployment of AI on IoT edge devices. Impact: Given that tea is a cornerstone of the domestic economy in regions like Bangladesh, an automated detection system for diseases like Blight, Red Rust, and Helopeltis has high practical and economic significance.

   Response:

   We deeply appreciate the reviewer for their positive comments regarding the relevance and significance of our research.

3. Literature Review Quality Comprehensiveness: The literature review covers the importance of tea, traditional detection challenges, and recent DL advances in crop disease. It correctly identifies a gap regarding the lack of large, standardized datasets in the tea industry. Missing Context: While it mentions advancements like attention mechanisms and wavelet transforms, more recent 2024-2025 SOTA comparisons in the specific tea domain could further strengthen the context.

   Response:

   We sincerely appreciate the reviewer for their constructive comments and for pointing out the opportunity to further strengthen our literature review. We entirely agree that incorporating more recent state-of-the-art comparisons provides a much

stronger contextual foundation for our research. In response to your highly valuable feedback, we have expanded our literature review to include recent 2024 and 2025 studies focusing specifically on deep learning advancements within the tea domain, including Vision Transformers and recent YOLOv8/YOLO11 variants. This expanded discussion highlights that while these modern architectures are powerful, they frequently face limitations regarding massive data requirements or struggles in simultaneously capturing local lesions and global structure. We then explicitly detailed how our dual-branch feature fusion approach directly addresses these exact limitations.

4. Methodology and Technical Soundness Clarity: The methodology is well-detailed, specifying a stratified 80-10-10 split , the use of the AdamW optimizer, and a custom multilayer perceptron (MLP) classification head. Assumptions: The use of ImageNet-based normalization and resizing to 224 X 224 pixels are standard and valid for these architectures. Reproducibility: The authors provide specific hyperparameters (initial learning rate of 0.001, batch size of 16) and have made their dataset and code publicly available, which is excellent for reproducibility.

Response:

We sincerely appreciate the reviewer for their positive evaluation of our methodology and for highlighting the technical soundness and reproducibility of our work.

5. Data, Experiments, and Results Datasets: The dataset is well-described, noting the use of an iPhone 12 Pro Max and expert annotation protocols to ensure ground-truth validity. Experiment Design: The benchmarking against six diverse architectures (ViT, ResNet50, etc.) is rigorous. Results: The hybrid model achieved 96.80% accuracy and a macro AUC of 0.9980. A notable finding is that while EfficientNetV2-B3 had slightly higher raw accuracy, the proposed hybrid framework showed better convergence stability.

Response:

We sincerely appreciate the reviewer for their thorough evaluation and highly positive assessment of our data collection, experimental design, and overall results.

6. Analysis and Discussion Insight: The discussion includes a cross-dataset generalization test on the teaLeafBD benchmark, which is a major strength. Limitations: The authors honestly acknowledge the relatively small dataset size (2,000 images) and the current lack of hardware-specific edge benchmarking.

Response:

We sincerely appreciate the reviewer for their positive feedback regarding our analysis and discussion.

7. Clarity, Structure, and Writing Quality Organization: The paper follows a logical flow from data acquisition to model evaluation. Language: The writing is formal and suitable for an international journal.

   Response:

   We sincerely appreciate the reviewer for their highly positive feedback regarding the clarity, structure, and writing quality of our manuscript.

8. Figures and Tables Quality: Figure 1 provides a clear overview of the methodology. Table 1 effectively summarizes the dataset attributes. Improvement: Ensure all axis labels in performance curves (e.g., Fig 11, Fig 12) are high-resolution and clearly readable in the final typeset.

   Response:

   We sincerely appreciate the reviewer for their positive feedback regarding our methodology overview and dataset summaries, as well as for the highly practical recommendation to improve the clarity of our performance curves.

9. Ethical and Reproducibility Considerations Ethics: The study involves plant materials in a natural setting; the authors state no competing interests and provide a data availability statement. Reproducibility: Strong, due to the public GitHub repository and detailed hyperparameter listing.

   Response:
   We sincerely appreciate the reviewer for their highly positive feedback regarding the ethical considerations and reproducibility of our study.

10. Strengths and Weaknesses Strengths: Novel dual-branch feature fusion approach. Publicly available, expert-annotated dataset. Cross-dataset validation demonstrating robustness. Weaknesses: Relatively small dataset size for deep learning (2,000 images). Absence of explainable AI (GradCAM) visualizations in the current version. Lack of empirical inference time on actual IoT hardware.

    Response:

    We sincerely appreciate the reviewer for their comprehensive summary of our study's strengths and weaknesses.

11. Detailed Reviewer Comments Major: The authors should provide a more in-depth comparison of the computational complexity (FLOPs/Parameters) between the hybrid model and the baseline models to truly justify "lightweight" claims. Expand the "Limitations" section to explicitly discuss how the model might handle multi-disease

leaves (co-infection), which is common in the field. Minor: Standardize the spelling of "EfficientNet" throughout the document (some instances have spaces). Ensure all citations in the text are consistently formatted according to PLOS One guidelines.

Response:

We sincerely appreciate the reviewer for their detailed and highly constructive feedback, which has significantly enhanced the technical rigor and overall polish of our manuscript. To provide a rigorous justification for our lightweight claims, we have calculated the total trainable parameters and FLOPs for all evaluated architectures. We have incorporated these computational complexity metrics into a newly established subsection and table within the Discussion section, alongside an expanded analysis comparing our hybrid framework's efficiency directly against the heavier baseline models. We also completely agree with your insightful observation regarding co-infections in natural agricultural settings. We have explicitly expanded the Limitations and Future Work section to discuss how the current model treats disease classifications as mutually exclusive, and we have outlined our future transition toward multi-label classification to accurately detect multi-disease leaves. Furthermore, we have meticulously reviewed the entire document to standardize the spelling of EfficientNet consistently without spaces, and we have verified that all in-text citations and bibliographic entries strictly adhere to the PLOS ONE formatting guidelines.

Reviewer #2

All corrections made in response to comments raised by Reviewer 1 are highlighted in yellow in the revised manuscript.

Most of the issues have been addressed. However, improvements can be made by including a flowchart diagram of the proposed hybrid technique and, in the paper (not as a supporting doc), the confusion matrix and classification report obtained by the proposed method. It is important for readers who want to compare it with their own work, etc.

Response:

We sincerely appreciate the reviewer for their constructive feedback and for highlighting how we can further improve the clarity and comparative utility of our manuscript. We completely agree that a visual flowchart greatly enhances the comprehensibility of our dual-branch architecture, and that providing the confusion matrix and classification report directly within the main text is essential for future researchers looking to benchmark their own work against our findings. We have designed a comprehensive flowchart diagram illustrating the complete data pipeline and architectural flow of our proposed hybrid technique. Furthermore, we have successfully moved the detailed confusion matrix and the comprehensive classification report from the supporting documents directly into the Models Evaluation Results section of the main manuscript.

Reviewer #3

All comments have been addressed. The paper is now improved and is clear and understandable for domain experts.

Response:

We are thrilled to hear that you find the manuscript significantly improved and readily understandable for domain experts, and we deeply thank you for your final endorsement of our research.
